# ^90^Y post-radioembolization clinical assessment with whole-body Biograph Vision Quadra PET/CT: image quality, tumor, liver and lung dosimetry

**DOI:** 10.1007/s00259-024-06650-9

**Published:** 2024-02-13

**Authors:** Konstantinos G. Zeimpekis, Lorenzo Mercolli, Maurizio Conti, Hasan Sari, Axel Rominger, Hendrik Rathke

**Affiliations:** 1 Department of Nuclear Medicine, Bern University Hospital, Inselspital, University of Bern, Freiburgstrasse 18, Bern, 3010 Switzerland; 2 Molecular Imaging, Siemens Healthineers, Knoxville, TN USA; 3 Advanced Clinical Imaging Technology, Siemens Healthcare AG, Lausanne, Switzerland

**Keywords:** Whole-body PET/CT, yttrium-90, Image quality, Dosimetry, Quadra

## Abstract

**Purpose:**

Evaluation of ^90^Y liver radioembolization post-treatment clinical data using a whole-body Biograph Vision Quadra PET/CT to investigate the potential of protocol optimization in terms of scan time and dosimetry.

**Methods:**

17 patients with hepatocellular carcinoma with median (IQR) injected activity 2393 (1348–3298) MBq were included. Pre-treatment dosimetry plan was based on ^99m^Tc-MAA SPECT/CT with Simplicit90Y™ and post-treatment validation with Quadra using Simplicit90Y™ and HERMIA independently. Regarding the image analysis, mean and peak SNR, the coefficient of variation (COV) and lesion-to-background ratio (LBR) were evaluated. For the post-treatment dosimetry validation, the mean tumor, whole liver and lung absorbed dose evaluation was performed using Simplicit90Y and HERMES. Images were reconstructed with 20-, 15-, 10-, 5- and 1- min sinograms with 2, 4, 6 and 8 iterations. Wilcoxon signed rank test was used to show statistical significance (*p* < 0.05).

**Results:**

There was no difference of statistical significance between 20- and 5- min reconstructed times for the peak SNR, COV and LBR. In addition, there was no difference of statistical significance between 20- and 1- min reconstructed times for all dosimetry metrics. Lung dosimetry showed consistently lower values than the expected. Tumor absorbed dose based on Simplicit90Y™ was similar to the expected while HERMES consistently underestimated significantly the measured tumor absorbed dose. Finally, there was no difference of statistical significance between expected and measured tumor, whole liver and lung dose for all reconstruction times.

**Conclusion:**

In this study we evaluated, in terms of image quality and dosimetry, whole-body PET clinical images of patients after having been treated with ^90^Y microspheres radioembolization for liver cancer. Compared to the 20-min standard scan, the simulated 5-min reconstructed images provided equal image peak SNR and noise behavior, while performing also similarly for post-treatment dosimetry of tumor, whole liver and lung absorbed doses.

**Supplementary Information:**

The online version contains supplementary material available at 10.1007/s00259-024-06650-9.

## Introduction

Hepatocellular carcinoma (HCC) is the sixth most commonly diagnosed cancer worldwide, with ever-rising mortality and incidence, and is the third leading cause of cancer-related deaths worldwide [1–3]. Although surgical resection and liver transplantation are still the best curative options for small HCC, the lack of transplanted organs and recent advances in targeted radionuclide therapy have changed the management of intermediate- and advanced- stage HCC [4]. The latter option includes glass or resin microspheres (size of µm), which contain yttrium-90 (^90^Y), a radionuclide that emits high-energy radiation in the form of electrons, placed with a catheter in the liver arteries near the tumor site, and flushed into the tumor itself by relatively higher blood perfusion in HCC. This radiation can locally deposit energy, causing cancer cell death with minimal damage to peripheral healthy liver tissue and lungs. This treatment method is called ^90^Y-radioembolization or Selective Internal Radiation Therapy (SIRT) and has been proven to be very efficient, providing high treatment efficacy for liver cancer during the last decade, with the potential to achieve complete remission in more than 80% of HCC patients. SIRT has been increasingly used to treat HCC patients [5–9]. In addition to HCC, intrahepatic cholangiocarcinoma (CCC) is the second most common cause of primary liver cancer and has an even higher mortality than HCC, although it has a significantly lower incidence rate [10, 11] and can also be treated with SIRT.

Single-photon emission computed tomography with computed tomography (SPECT/CT), `based on technetium-99m (^99m^Tc) macro aggregated albumin (MAA), is used for the pre-treatment dosimetry plan and is regarded as the standard for post-treatment dosimetry validation [12, 13]. However, ^90^Y Bremsstrahlung SPECT imaging presents certain challenges [12, 14, 15] such as dominant photon scatter, collimator septal penetration, and limited spatial resolution [16]. The post-treatment dosimetry holds significance in determining whether the desired absorbed dose at the tumor site has been achieved. Additionally, it aids in evaluating the treatment success through follow-up scans, which demonstrate tumor regression.

Apart from the predominantly emitted beta particles, a rare decay path of ^90^Y can result in an excited state of zirconium-90 (^90^Zr), emitting a 2 MeV photon, which can then produce electron-positron pairs. Modern positron emission tomography with computed tomography scanners (PET/CTs) can overcome the limitations arising from the low count statistics of the extraordinarily small branching ratio (32 ppm) [17] owing to their overall higher sensitivity performance. Specifically, for post-treatment dosimetry evaluation, which is important for assessing the outcome of irradiation of tumor lesions, PET has been gaining favor because of its higher spatial resolution and quantitative accuracy compared to SPECT [12, 14, 15, 18–20]. The increased use of PET for SIRT imaging has been made possible by the introduction of TOF PET [18, 21], which reduces the variance in the images by a factor that is proportional to the size of the object and inversely proportional to the time resolution of the PET scanner [21].

With the advent of whole-body PET/CT, which covers most of the body within one scan, a new interest in PET dosimetry is available, since a direct assessment of lung dose and healthy liver tissue (both organs at risk, as higher doses can lead to radiation pneumonitis or liver malfunction) is possible after treatment. Furthermore, the scan times can be dramatically reduced, facilitating patient comfort and overall clinical throughput. With the Biograph Vision Quadra (Siemens Healthineers, Knoxville, TN, USA, “Quadra”), we have previously conducted the first body phantom study with ^90^Y [22] to test its performance regarding image quality and quantitation. Based on this study, the clinical ^90^Y PET protocol was optimized to achieve the best image quality with the shortest possible scan time. Biograph Vision Quadra provides one of the highest overall sensitivities of all available PET/CT scanners in the market [23], providing images of all important organs in the torso within one scan (head to upper thighs) with high image quality (low noise) acquired in significantly shorter scan times than usual (~ 50%) [22].

However, while there are publications that show an agreement between pre- and post-treatment dosimetry [24], it has been shown that the pre-treatment plan can suffer from large variations, and the absorbed doses can be under- or overestimated [25]. Even in publications that show agreement in terms of statistical significance, the mean absorbed doses can vary in absolute values by many Grays (Gy), and the pre-treatment plan tends to underestimate the tumor dose. However, there is no consensus regarding the medical interpretation of tumor sustainability, liver function, and overall patient outcome [26–29]. This is where the necessity for standardization of post-treatment PET dosimetry becomes imperative, as there are also many clinical factors affecting the outcome and efficacy of treatment that have not been investigated or fully explored. There is an increasing demand for standardization of post-treatment PET dosimetry validation.

The purpose of this study was threefold: first, to measure the actual absorbed dose with a post-treatment PET scan and compare it to expected dose based on the pretreatment plan. Second, to investigate the feasibility of as short scans as possible, compared to the standard 20-min long scans of today’s clinical routine by retaining however equivalent image quality. Third, to perform also lung dosimetry. The advantage for the patient is that a further scan is not required like in SPECT, since with the whole-body PET, the lungs are within the acquired axial field-of-view (AFOV).

## Materials and methods

### Scanner

All patients underwent a scan on a Quadra PET/CT scanner which employs silicon photomultiplier-based detectors with 3.2 × 3.2 × 20 mm^3^ lutetium-oxoorthosilicate crystals [30]. Quadra is comprised of 32 detector rings, each with 38 detector blocks, which provide an AFOV of 106 cm. The data were acquired using the complete AFOV with a maximum ring distance of 322 crystals (MRD 322). The reconstruction algorithm using the data acquired with the full FOV (MRD 322) is called ultra-high sensitivity (UHS). The overall system sensitivity is 176 cps/kBq for the UHS, whereas the time-of-flight (TOF) is 230ps [30]. The attenuation correction was based on the CT data. Randoms correction was applied employing the delayed event subtraction method. For the scatter correction a novel 3D correction algorithm was used, which estimates the full 3D scatter profile from the residual between measured and modeled data [31]. More specifically, the 2D measured data together with the 2D single scatter simulation (2D-SSS) model-based scatter provide the non-scattered true image estimate. Spatial resolution and slice thickness was 1.65 mm.

### Patient population

In this retrospective study, the patient scans were performed between March 2021 and September 2022. 17 patients (median age, 71 years; interquartile range (IQR) 64–78) were included. Glass microspheres (TheraSphere®; Boston Scientific, Marlborough, MA, USA) were used for the transarterial radioembolization (TARE) [32]. The average time between the intervention and imaging scan was 3.1 ± 0.5 h. There were 13 male and 4 female patients: 11 diagnosed with HCC, 2 with CCC, 2 with both HCC and CCC, and 2 with hepatic metastasis. The tumors were localized in the right lobe in 15 cases and in the left lobe in 2 cases. There was no minimum tumor volume requirement applied, because all patients had sufficiently large tumors (well over 1 cm). For 14 patients there was one solid tumor on one location whereas for 3 patients there were at least 2 tumor sites. In the analysis we considered the tumors with the highest uptake of Y90, based on the tumor segmentations that was done by the responsible physician during pretreatment planning. The mean injected ^90^Y activity and its standard deviation (SD) was 2502 ± 1272 MBq (range 181–6198 MBq, median 2393 MBq, IQR 1348–3298).

### Preparation and measurement of the Y-90 microsphere’ activity

The activity was measured using a well-type dose calibrator (ISOMED 2010). The calibrator satisfied the Swiss regulatory requirements and was calibrated by the Swiss Federal Institute of Metrology (METAS) [33]. The application took place at the Angiology Department in the presence of a radiologist and a nuclear medicine physician. Once the intervention was completed, the patients were transported to the Nuclear Medicine Department for the PET/CT scan.

### Pre-treatment SPECT plan

For the pre-treatment dosimetry plan, according to the EANM guidelines [34] a ^99m^Tc-MAA scan that can depict the tumor sites in the liver based on the percentage uptake. Lung shunt ratio is the geometric mean between the counts measured in the whole liver and the measured counts in the lung. The lung is one of the most radiosensitive organs in the body and the absorbed dose must be kept below 20 Gy due to possible radio-pneumonitis. MAA scan provides a prediction of the ^90^Y microsphere distributions. Differences can arise which is subject of discussion especially for resin microspheres. Nonetheless this is the actual clinical standard of care.

Protocol of the technetium scan consists of a ventral and dorsal planar imaging 15 min in total. The quantitative SPECT scan is 20 min long, reconstructed with 8 iterations 4 subsets, and a reconstruction matrix of 256 × 256. The CT for attenuation correction has the following parameters: 110 kV, modulated mA, slice thickness 2 mm, 256 × 256 reconstruction matrix. The SPECT and CT images are loaded on Simplicit90Y along with the planar images with the segmentation of liver and lung, which are used to calculate the lung shunt ratio. The segmentation of the whole liver (right and left lobe), perfused tumor and viable perfused tumor site are performed on the CT images by the responsible physician.

### Post-treatment PET scan

The patients were positioned on the bed at the center of the scanner. A helical CT scan was acquired for the PET attenuation correction with the following parameters: 80 kV tube voltage, 39 modulated mAs tube current, 38.4 mm total collimation width, 5 mm slice thickness and a pitch 0.8. The reconstruction matrix for the CT images was 512 × 512, with 644 slices, and the voxel dimensions were 1.523 × 1.523 × 1.6 mm^3^. Following CT, PET acquisition was performed with a total acquisition time of 20 min. The AFOV covered the body area from the head to thighs. The voxel dimensions were 3.3 × 3.3 × 1.65 mm^3^. The scanner was calibrated for ^90^Y by the manufacturer, and a calibration factor was used to normalize the data. The data were decay-corrected to the injection time.

### Reconstruction

All data were reconstructed using TOF, point spread function (PSF) recovery, and 3D ordered subset expectation maximization (3D-OSEM). Subsequently, the list-mode data were reconstructed using different parameters. The images were generated with iteration numbers 2, 4, 6, and 8 with 5 subsets, a gaussian filter of 2 mm full width at half maximum (FWHM), and a matrix size of 220 × 220 with UHS, based on the results of a phantom evaluation by another study [22]. Furthermore, images were generated, except for the original acquisition time of 20 min, using the first 15-, 10-, 5- and 1-min of the list-mode data.

### Image quality analysis

The image quality was evaluated in terms of the peak and mean signal-to-noise ratio (SNR_peak_, SNR_mean_), coefficient of variation (COV) that shows the behavior of image noise, and lesion-to-background ratio (LBR). The evaluation was performed independently on both syngo.via (Siemens Healthineers, Chicago, IL, USA) and MATLAB after manual segmentation using itk-SNAP an open-source software [35]. Spherical volume-of-interest (VOI) masks (of variable size for each patient) were manually drawn on the 20-min PET images, centered on the lesion with the highest uptake (VOI_L_). On the same slice, 8 VOIs of the same size were drawn on the background, that is, in areas without any uptake. Henceforth, this will be referred to as VOI_BG_ and will denote the average of all 8 background VOI means combined. The maximum, peak, mean, and SD values in kBq/ml were extracted. The VOIs were then propagated to all image datasets. For every patient, there were 20 image datasets (5 reconstruction times by 4 different iteration numbers).

The peak SNR was measured as the difference of the average over a cubic centimeter volume around the voxel of the VOI_L_ with the maximum signal (VOI_L−peak_) and the mean of the background VOI (${\text{VOI}}_{\text{BG-mean}})$, to the SD of the background VOI (VOI_B−SD_), as follows:

$${\text{SNR}}_{\text{peak}}\text{= }\frac{{\text{VOI}}_{\text{L-peak}}- {\text{VOI}}_{\text{BG-mean}}}{{\text{VOI}}_{\text{BG-SD}}}$$

The SNR_mean_ was calculated as the ratio of the difference between the measured mean activity concentration between VOI_L_ and VOI_B_ to the SD of the VOI_B_ as follows:

$${\text{SNR}}_{\text{mean}}\text{= }\frac{{\text{VOI}}_{\text{L-mean}}- {\text{VOI}}_{\text{BG-mean}}}{{\text{VOI}}_{\text{BG-SD}}}$$

The COV was calculated as the ratio of the image background noise (VOI_BG−SD_) to the mean of the background VOI (VOI_BG−mean_), as follows:

$$\text{COV= }\frac{{\text{VOI}}_{\text{B-SD}}}{{\text{VOI}}_{\text{B-mean}}}$$

Finally, the lesion-to-background ratio was calculated as the ratio of the maximum voxel value (VOI_L−max_) to the mean of the background VOI (VOI_BG−mean_).

$$\text{LBR = }\frac{{\text{VOI}}_{\text{L-max}}}{{\text{VOI}}_{\text{BG-}\text{mean}}}$$

The evaluated metrics were presented as the average of all 17 patients. The results in the figures were presented in the form of boxplots denoted by the median (vertical line), mean (yellow marker), interquartile range and minimum and maximum whiskers defined as 1.5 * IQR. The outliers were also shown (white markers). The inter-comparison between the different reconstruction times of the 2 iterations were also shown on the figures in terms of the Wilcoxon p-value for statistical significance. For the 20 min, a comparison between the different number of iterations was presented too. The results were presented as median (IQR).

### Dosimetric analysis

The pre-treatment dosimetry plan was based on Simplicit90Y™, a commercial dosimetry software (Mirada Medical Ltd, Oxford, UK; Boston Scientific Corporation, Marlborough, MA, USA) [36]. Simplicit90Y™ relies on a local deposition model for dose calculations [37]. The multi-compartment algorithm estimates the relative absorbed dose of the perfused volume, tumor, and whole liver normal tissue by considering the number of counts within each segmented anatomical volume on the SPECT/CT image. The mean lung dose was estimated based on the geometric mean method and by estimating the lung shunt ratio between the lungs and liver on anterior and posterior planar images.

For the post-treatment dosimetry validation, the PET images were loaded on both Simplicit90Y™ and HERMIA GOLD Smart Workstation 2.17 (Hermes Medical Solutions AB, Stockholm, Sweden) using the Voxel Dosimetry 1.1 toolbox [38, 39]. Hermes has a voxel dosimetry approach, that involves the so called semi-Monte Carlo (sMC) algorithm to produce the dose calculations, for that reason, as input it requires quantitative PET images. This sMC method is proposed to produce the calculations much faster compared to the time consuming full Monte Carlo. The physics behind the sMC is the following: the electron and photon transport are individually considered. The electron energy is assumed to be absorbed locally, i.e. in the same voxel where the decay has happened, wherea a point-wise transport is used for the photon energy deposition. Thus, we could independently evaluate the performance of both software platforms that utilize different approaches.

The reconstruction with 20 min and 2 iterations was taken as the reference for the post-treatment dosimetry. The p-values in parentheses in the dosimetry section concerned comparison between 20- and 15-, 15- and 10-, 10- and 5-, 5- and 1-, 20- and 5- and finally 20- and 1- min reconstruction times. The comparisons are described in terms of predicted (pre-treatment SPECT) and actual (post-treatment PET) absorbed doses.

The main values from the dose-volume histograms (DVH) were extracted, i.e., D_2_, D_50_, and D_70_, each of which indicating the absorbed dose delivered to 2%, 50%, and 70% of the tumor volume. D_mean_ denotes the mean absorbed dose delivered to the whole tumor volume. The whole liver normal tissue absorbed dose and mean lung dose (based only on HERMIA) were also extracted.

### Statistical analysis

The non-parametric Wilcoxon signed rank test for paired data was used because data normality could not be assumed due to the small size of the sample to assess the difference of statistical significance of the mean values of all evaluated metrics. This test is unaffected by outliers. A p-value < 0.05 was considered as statistical significant. The statistical testing was performed using the statsmodels package of Python (Python, version 3.12).

In order to keep the results section comprehensible the p-values are shown only on the diagrams.

## Results

For all comparisons between iteration numbers, the 2 iterations provide the best outcome and with increasing number of iterations up to 8, there is a progressive degradation for all metrics. This is also shown on the figures.

**Fig. 1:**
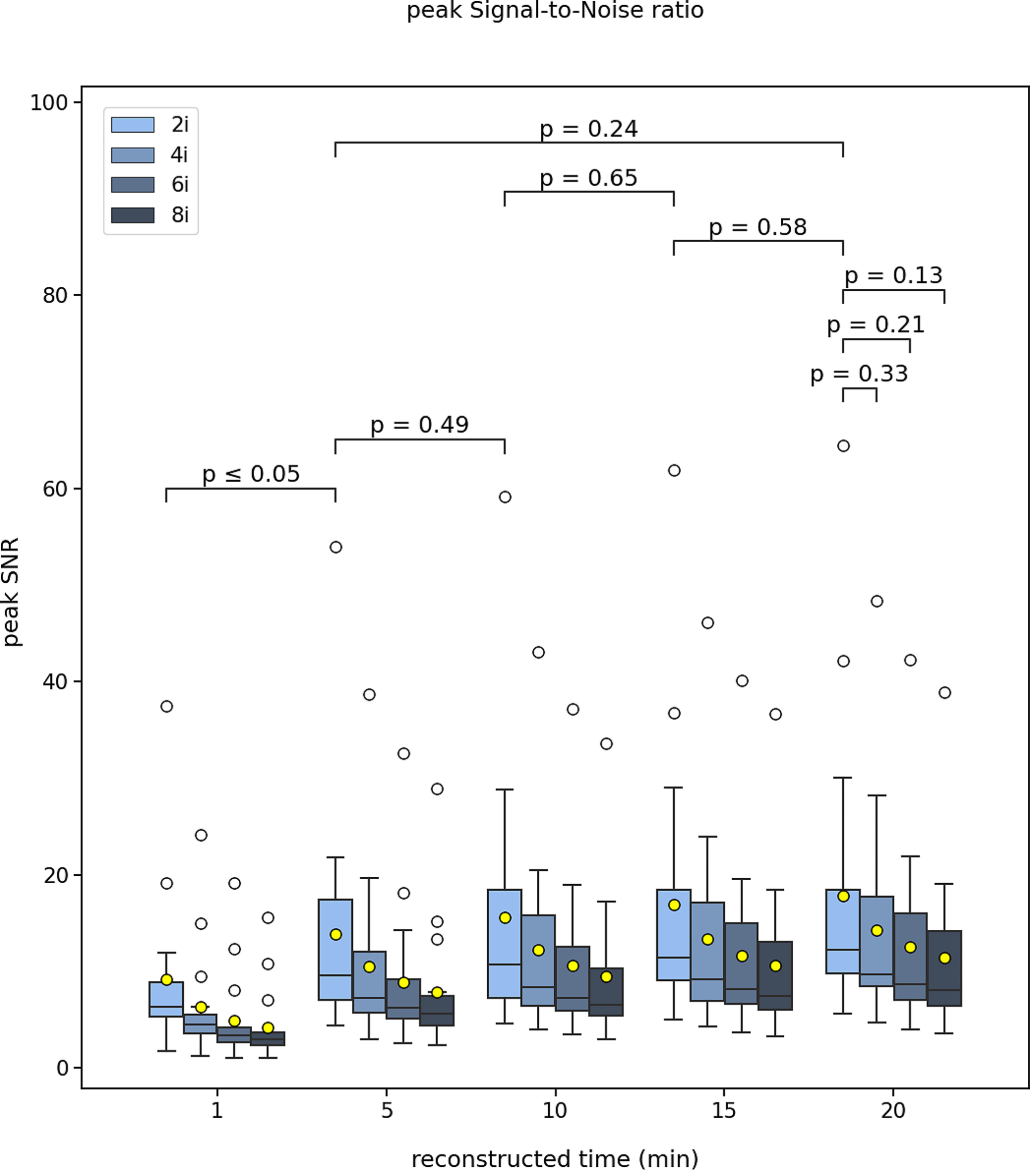
Boxplot of the image peak SNR for all 17 patients scanned post-^90^Y-radioembolization for various reconstruction times (20, 15, 10, 5, 1 min) and different number of iterations (2, 4, 6, 8). The mean (yellow mark) and median (black horizontal line) are depicted along with outliers (white marks). The p-values of the Wilcoxon test are depicted for comparison between the different reconstruction times for 2 iterations and for the 20 min between the 2, 4, 6, and 8 iterations

The peak SNR did not show any difference between 20- and 15-, 15- and 10-, 10- and 5- min reconstruction times, but showed a significant difference between 5- and 1-min (Fig. 1). The median (IQR) peak SNR was measured 12.3 (8.1–21.0), 11.5 (7.8–21.0), 9.6 (6.8–20.9), 9.2 (6.6–18.3) and 6.4 (5.2–9.1) for the 20-, 15-, 10-, 5- and 1- min reconstruction times. There was no difference between 2 and 4, 6 and 8 iterations.

Likewise, the median (IQR) mean SNR was measured 14.3 (11.4–27.5), 13.4 (9.6–26.7), 11.9 (9.2–26.7), 10.9 (8.0-25.5) and 9.6 (7.0-14.8), and did not show any difference, in contrast to the peak SNR, between 20- and 15-, 15- and 10-, 10- and 05-, 05- and 01, and 20- and 5-min reconstruction times (*p* = 0.92, 0.78, 0.66, 0.98, 0.49). No difference was shown between the different number of iterations as well (*p* = 0.98, 0.87, 0.82).

**Fig. 2:**
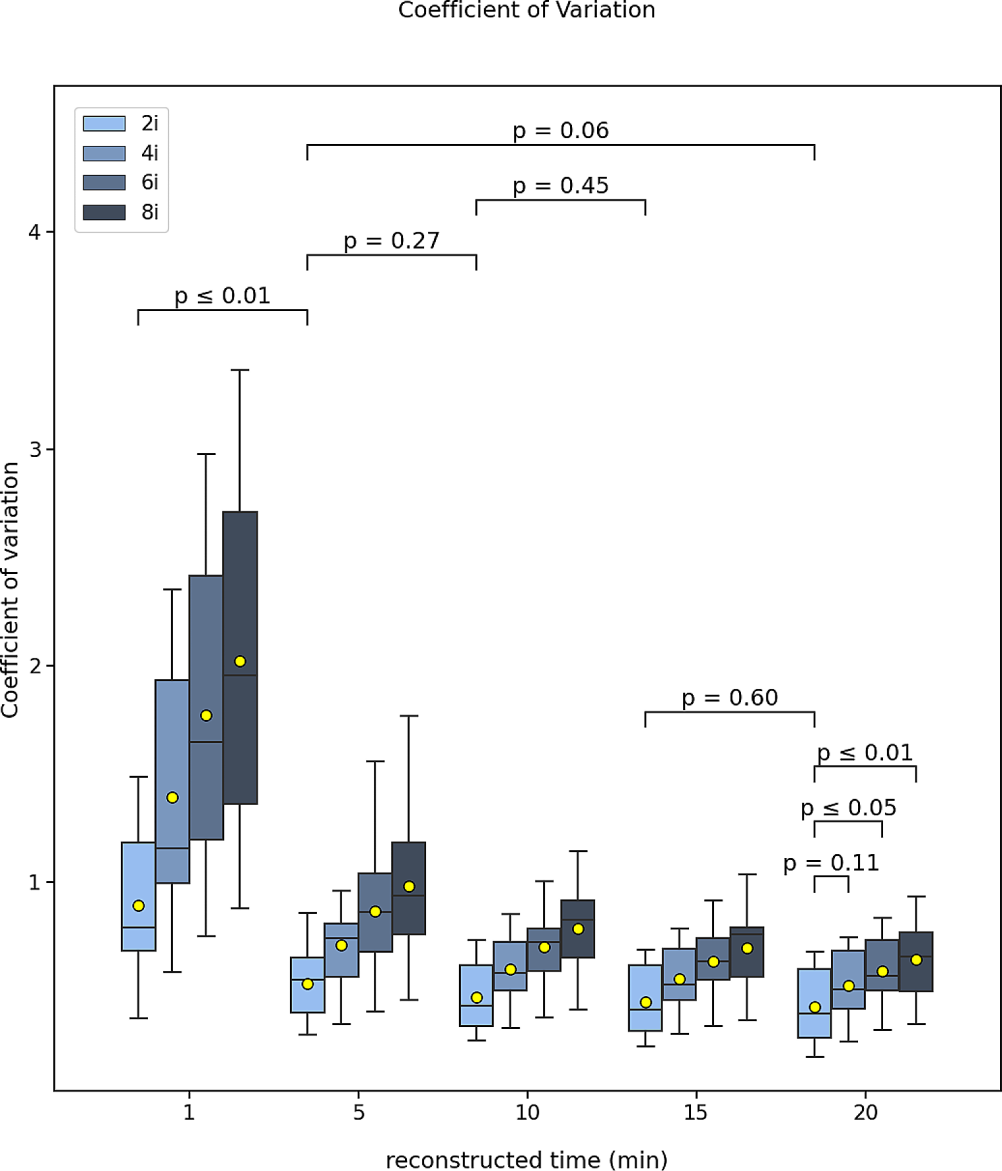
Boxplot of the image coefficient of variation for all 17 patients scanned post-^90^Y-radioembolization for various reconstruction times (20, 15, 10, 5, 1 min) and different number of iterations (2, 4, 6, 8). The mean (yellow mark) and median (black horizontal line) are depicted along with outliers (white marks). The p-values of the Wilcoxon test are depicted for comparison between the different reconstruction times for 2 iterations and for the 20 min between the 2, 4, 6, and 8 iterations

The coefficient of variation (COV) did not show any difference between 20- and 15-, 15- and 10-, 10- and 5- min reconstruction times, however, a difference was observed between 5- and 1- min (Fig. 2). The measured median (IQR) COV values were 0.40 (0.28–0.60), 0.41 (0.31–0.62), 0.43 (0.33–0.62), 0.56 (0.38–0.67) and 0.79 (0.65–1.21) for the 20-, 15-, 10-, 5- and 1- min reconstruction times. There was also a difference between 2 and 6, 8 iterations, but none with 4 iterations.

**Fig. 3:**
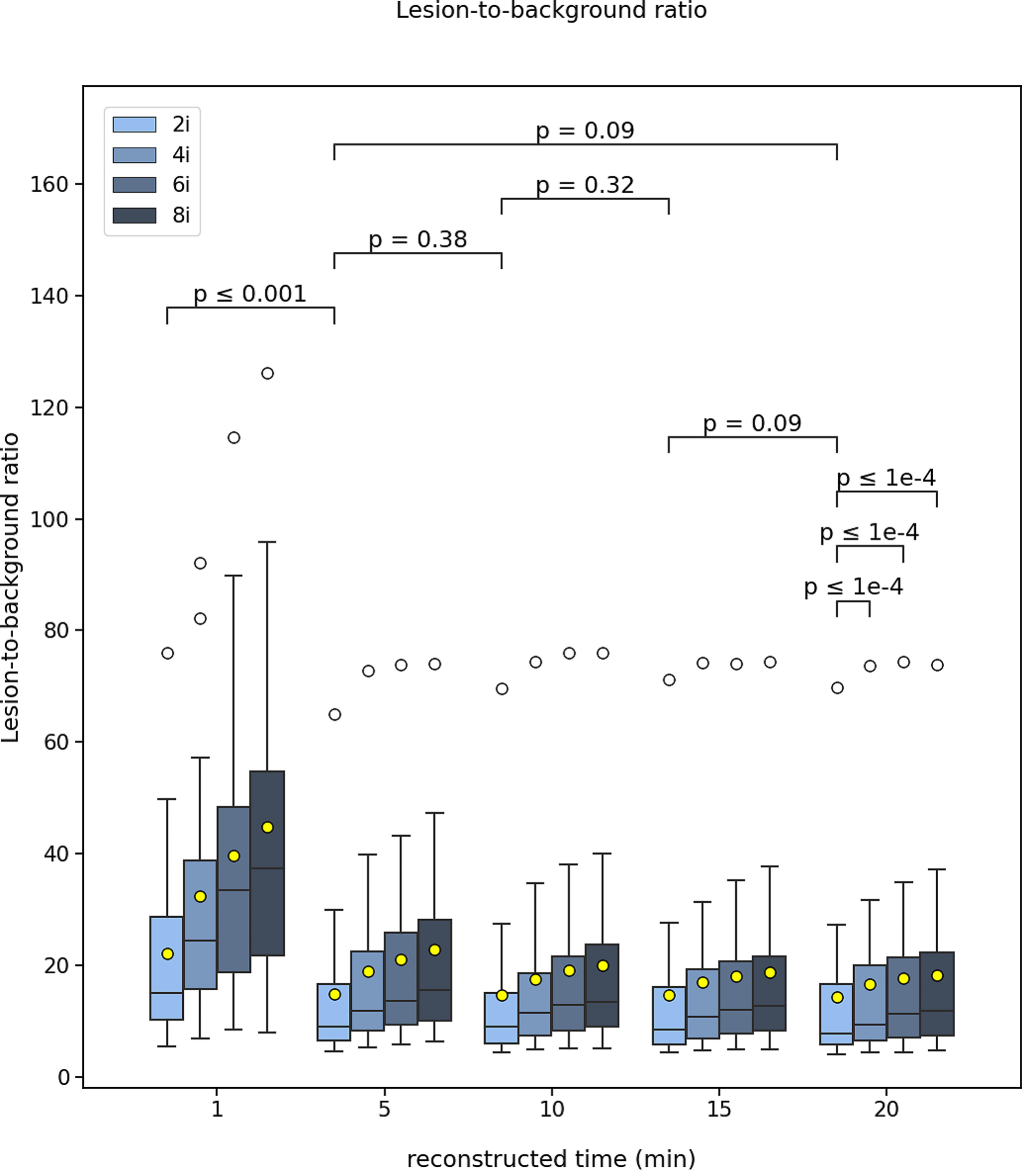
Boxplot of the image lesion-to-background ratio for all 17 patients scanned post-^90^Y-radioembolization for various reconstruction times (20, 15, 10, 5, 1 min) and different number of iterations (2, 4, 6, 8). The mean (yellow mark) and median (black horizontal line) are depicted along with outliers (white marks). The p-values of the Wilcoxon test are depicted for comparison between the different reconstruction times for 2 iterations and for the 20 min between the 2, 4, 6, and 8 iterations

Similarly, for the lesion-to-background ratio, there was no difference between the 20- and 15-, 15- and 10-, 10- and 5- min, and 20- and 5- min reconstruction times, but there was a difference between 5- and 1- min (Fig. 3). There was a difference between the 2 and 4, 6, 8 iterations. The median (IQR) lesion-to-background ratios were measured 7.8 (5.6–17.6), 8.6 (5.8–17.9), 9.1 (5.8–16.4), 10.8 (6.3–16.9), 15.0 (9.4–31.1) for the 20-, 15-, 10-, 5- and 1- min reconstruction times.

Regarding the dosimetry validation on Simplicit90Y™ the average predicted and actual mean tumor absorbed dose (D_mean_) based on the pre-treatment ^99m^Tc-MAA SPECT/CT scan and ^90^Y PET/CT were 299.8 ± 78.8 Gy (median 304.5 Gy, IQR 270.2-357.3) and 275.2 ± 139.4 Gy (median 279.0 Gy, IQR 135.9-364.9) (*p* = 0.6), respectively. Based on HERMIA it was 181.4 ± 65.5 Gy (median 204.6 Gy, IQR 137.8-233.6) (*p* ≤ 0.001). There was no difference between the predicted and actual mean tumor absorbed dose based on Simplicit90Y™ but a difference was shown with HERMIA.

**Fig. 4:**
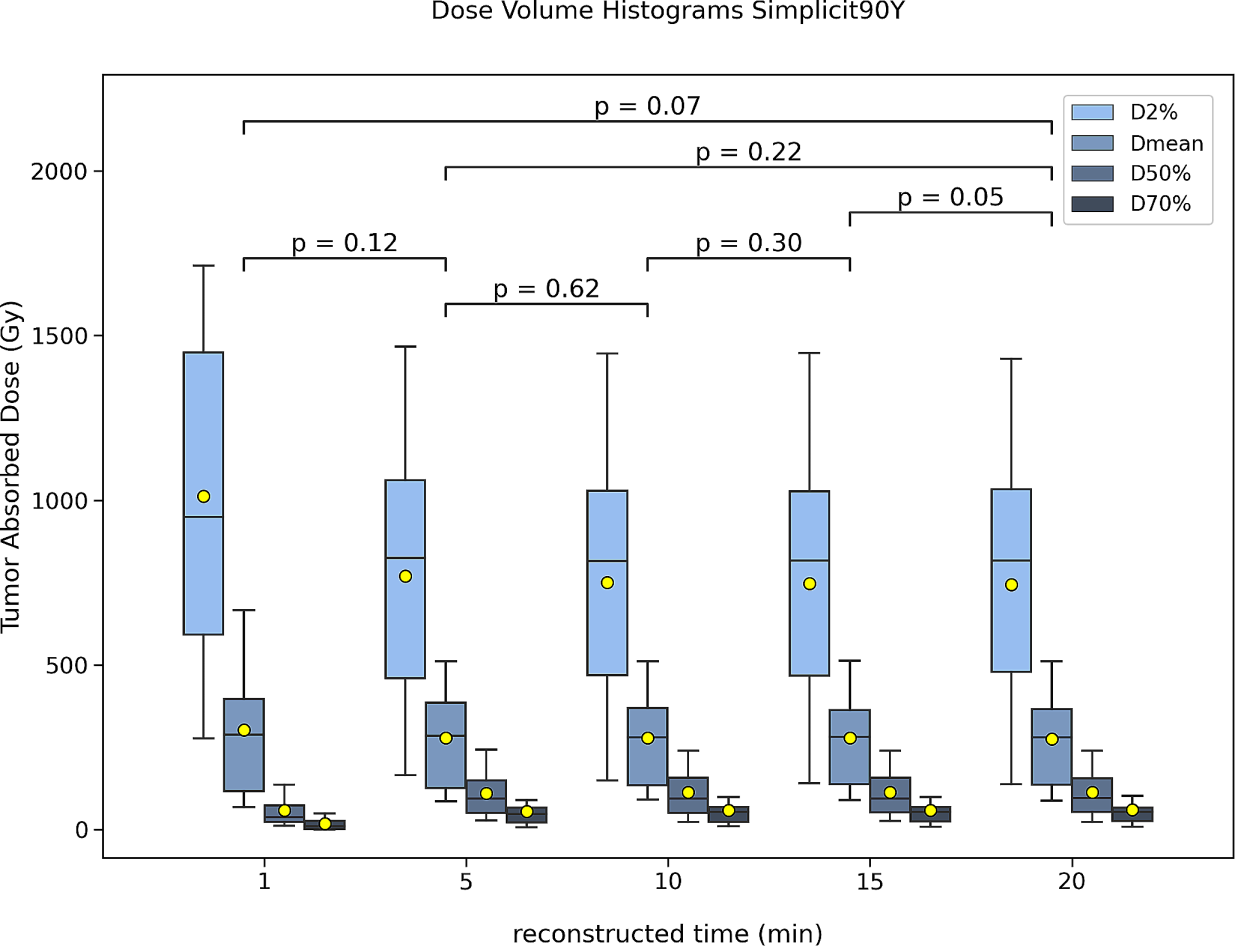
Boxplot of the mean lung absorbed dose averaged for all 17 patients scanned post-^90^Y-radioembolization for various reconstruction times (20, 15, 10, 5, 1 min) and different number of iterations (2, 4, 6, 8). The mean (yellow mark) and median (black horizontal line) are depicted along with outliers (white marks). The p-values of the Wilcoxon test are depicted for comparison between the different reconstruction times for 2 iterations and for the 20 min between the 2, 4, 6, and 8 iterations

Concerning the different reconstruction times, the average mean tumor absorbed doses on Simplicit90Y™ were 275.2 ± 139.4 Gy, 278.2 ± 141.6 Gy, 277.6 ± 141.0 Gy, 278.6 ± 142.0 Gy, 302.5 ± 182.5 Gy, for the 20-, 15-, 10-, 5- and 1- min reconstruction times (*p* = 0.05, *p* = 0.30, *p* = 0.62, *p* = 0.12, *p* = 0.22, *p* = 0.07), respectively. Similarly for HERMIA, they were 181.4 ± 65.5 Gy, 182.5 ± 65.4 Gy, 180.5 ± 64.6 Gy, 177.7 ± 62.5 Gy, 171.8 ± 62.7 Gy (*p* = 0.50, *p* = 0.16, *p* = 0.16 *p* = 0.16, *p* = 0.16, *p* = 0.09). For both dosimetry evaluations, there was no difference between all reconstruction times, from 20- down to 1- min reconstruction times (Fig. 4). In addition, there was no difference of statistical significance between 2 and 4, 6, 8 iterations.On Fig. 4, the trend of the dose volume histograms was depicted based on the dose absorbed by the following percentage tumor volumes 2%, 50%, 70%. The D_mean_ seems stable for all reconstruction times while D_50_ and D_70_ were lower on the 1- min scan. D_2_ presents the maximum spread on the 1-min scan and higher absorbed dose due to excessive noise.

The predicted and actual average mean whole liver normal tissue absorbed dose on the pre-treatment ^99m^Tc-MAA SPECT/CT scan and ^90^Y PET/CT were 36.2 ± 15.6 Gy (median 39.5 Gy, IQR 23.5–49.1) and 36.5 ± 15.2 Gy (median 38.5 Gy, IQR 30.2–44.7) (*p* = 0.96), respectively. For the 20-, 15-, 10-, 5- and 1- min reconstruction times the values were 36.2 ± 15.6 Gy, 36.4 ± 15.2 Gy, 36.4 ± 15.2 Gy, 36.4 ± 15.2 Gy and 33.7 ± 15.9 Gy, respectively. There was neither a difference between the predicted and actual average whole liver absorbed dose nor a difference between the reconstructed times. Moreover, no difference was observed between 2 and 4, 6, 8 iterations.

Finally, the predicted and actual average mean lung tissue absorbed dose on the pre-treatment ^99m^Tc-MAA SPECT/CT scan and ^90^Y PET/CT were 2.5 ± 2.0 Gy (median 2.3 Gy, IQR 0.9–3.1) and 2.0 ± 1.8 Gy (median 1.2 Gy, IQR 0.8–2.6), respectively. Between the predicted and actual average mean lung dose there was no difference of statistical significance.

**Fig. 5:**
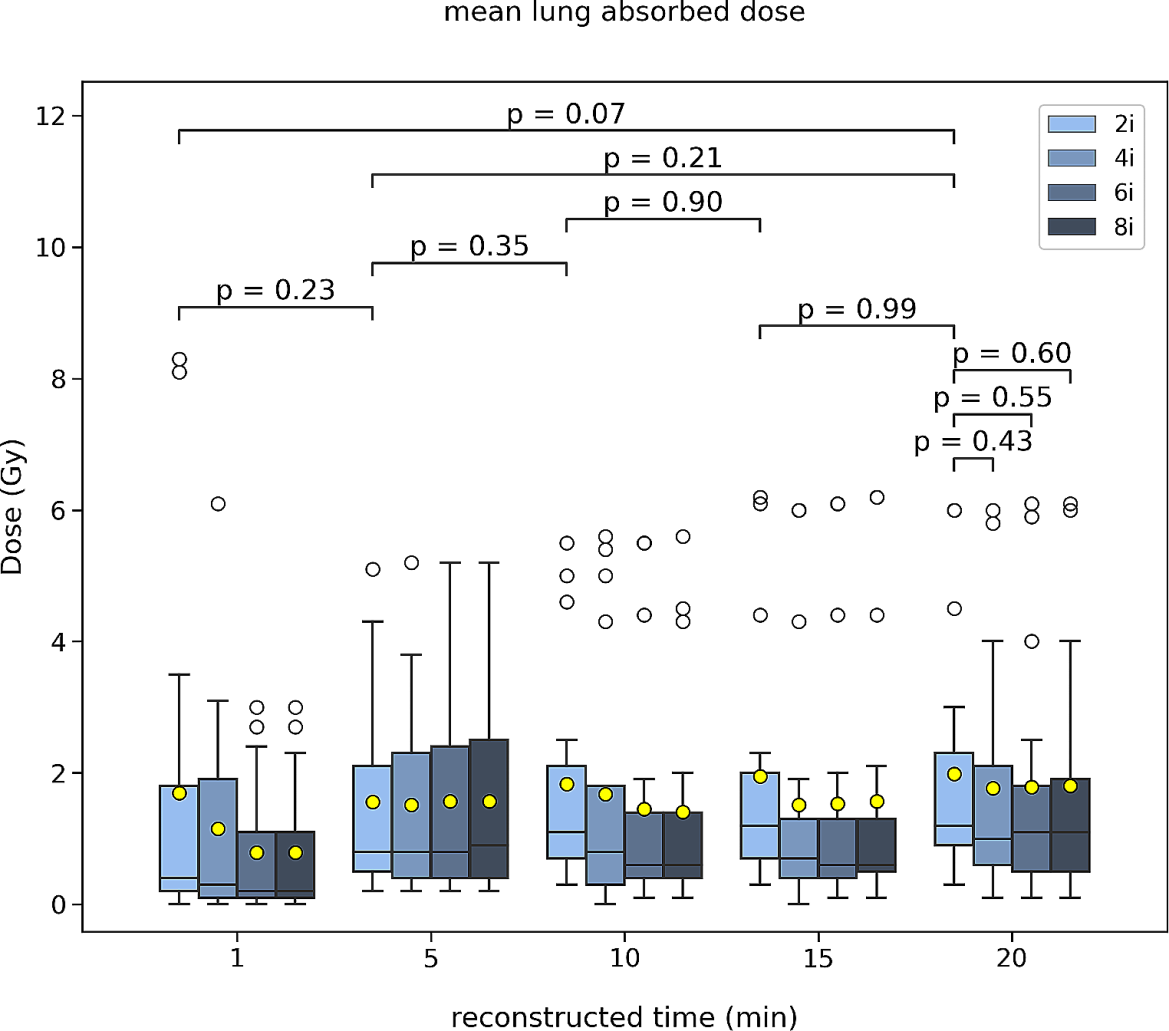
Boxplot of the dose volume histograms (D_2_, D_mean_, D_50_, D_70_) averaged for all 17 patients scanned post-^90^Y-radioembolization for various reconstruction times (20, 15, 10, 5, 1 min) and different number of iterations (2, 4, 6, 8). The mean (yellow mark) and median (black horizontal line) are depicted along with outliers (white marks). The p-values of the Wilcoxon test are depicted for comparison between the different reconstruction times for 2 iterations and for the 20 min between the 2, 4, 6, and 8 iterations

For the 20-, 15-, 10-, 5- and 1- min reconstruction times the values were 2.0 ± 1.8 Gy, 2.0 ± 1.8 Gy, 1.8 ± 1.6 Gy, 1.6 ± 1.5 Gy, 1.7 ± 2.6 Gy, respectively (Fig. 5). There was neither a difference of statistical significance between all reconstruction times nor between 2 and 4, 6, 8 iterations. In addition, for the patient with the maximum predicted mean lung dose (8 Gy), the actual was 3 Gy. Specifically, the mean left lung absorbed dose was 1.7 ± 2.5 Gy (median 0.9 Gy, IQR 0.3–1.5) and the mean right lung absorbed dose was 2.2 ± 2.7 Gy (median 0.8 Gy, IQR 0.4–3.3).

**Fig. 6:**
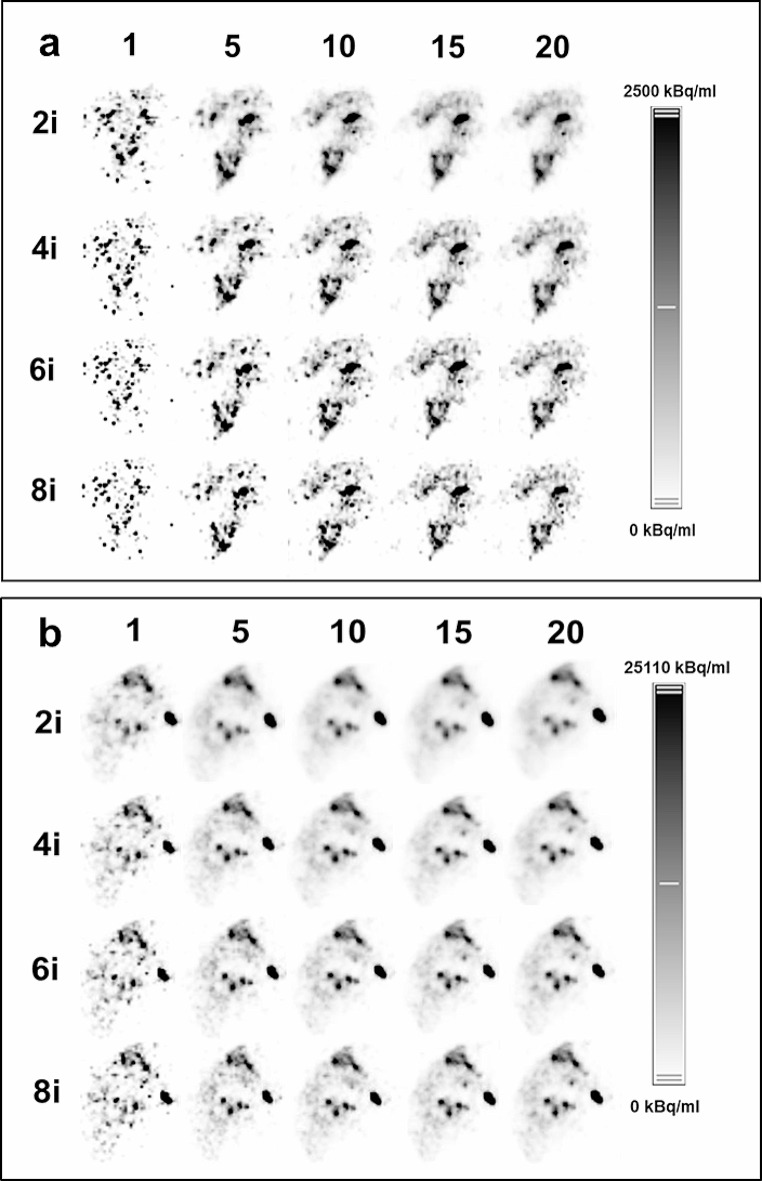
Clinical whole-body PET images of two patients post ^90^Y liver radioembolization injected with the lowest (**a**) and highest (**b**) activities with variable reconstruction times (20, 15, 10, 5, 1 min) and different number of iterations (2, 4, 6, 8). (**a**) Patient was injected with 181 MBq while in (**b**) with 6198 MBq

Figure 6 shows an PET clinical images depending on count statistics and number of iterations. Figure 6a shows the patient images with the lowest injected activity 181 MBq (max. uptake image concentration 5207 kBq/ml) and 6b with the highest injected activity of 6198 MBq (likewise 227,117 kBq/ml). In both examples, the 5-min reconstruction is still comparable to the original 20 min. It is also evident that with increasing number of iterations, the convergence starts to produce higher noise in the images. For the highest activity even with the 1 min scan, the localaization of the Y90 microspheres is pretty clear at the expense of negligible noise increase.

Qualitatively, in general the 1-min reconstructions showed more prominent noise and suboptimal delineation of the tumor uptake profile. Still for the majority of patients the images were not non-acceptable. Furthermore, 2 and 4 iterations looked more consistent compared to the more noisy images of the 6 and 8 iterations.

## Discussion

Qualitatively there is a consistency for all image quality metrics (peak SNR, COV and LBR) for 20 min down to 5 min. Practically this means that scans with only 5 min acquisition time are sufficient to produce the same image quality as for 20 min. This would improve further the patient comfortability and throughput for the daily clinical routine. The higher sensitivity of the Quadra facilitates the improvement of image quality by providing low-noise images at 5 min even with the sub-percentage positron emission fraction of ^90^Y. The peak SNR for the 20-, 15-, 10-, 5- and 1-min reconstruction times shares the same trend for the COV and LBR. We should note here that the injected activity, patient mass as well as geometry and location of tumor site varies heavily, as expected. Having stated that, we presented here only the averaging of all 17 patients, so for patients with higher injected activity and lower patient mass the image quality could be acceptable even at 1-min, on the other hand heavier patients with less injected activity could provide equal SNR at least with 5 min. Here we only want to present the clinical potential of the scanner and setting a precedent for protocol optimization.

As far as dosimetry is concerned, Simplicit90Y™ is a multi-compartmental MIRD image based dosimetry software, that assigns a homogeneous factor to convert kBq/ml to absorbed dose (Gy) to the whole segmented anatomical tumor according to the local deposition model. The absorbed dose is automatically provided based on the relative number of counts within the SPECT or PET image. In general, Simplicit90Y™ performed well and the actual tumor absorbed dose was no different than the predicted. Lower tumor absorbed dose, could be in general attributed to suboptimal pre-treatment tumor segmentation and poor tumor targeting, including also necrotic tissue within the perfused tumor volume, suboptimal injection of the activity, and longer latency period between planning and treatment session (longer than 2 weeks) that allows for tumor progression.

HERMIA’s voxel dosimetry module relies on the semi Monte Carlo, which transports the photons as in a full Monte Carlo simulation but deposits the electron/positron energy directly in the source voxel [38]. Therefore, the activity distribution in the PET image is taken as the source for the voxel-based dose calculation, making it susceptible to variations in the imaged ^90^Y distribution. Hermes seems to consistently exhibit lower absorbed doses as another recent phantom study has already shown [40]. This could be interpreted, in the basis of image-dependent introduction of errors like mis-registration between anatomical and functional segmented areas and more importantly due to signal spill over between smaller pixels in PET images (2–3 mm) and ^90^Y electron range (maximum range of 11 mm in soft tissue).

The post-treatment dosimetry showed that 6 patients had a higher tumor absorbed dose compared to the predicted (mean difference increase + 26%, range 11–36%) and 11 patients lower than predicted (mean difference decrease − 29%, range 5–79%). In both cases the whole liver normal tissue and lung absorbed dose were still below the accepted limits and consistent to the predicted dose.

It is already established, that the pre-treatment dosimetry plan of the ^90^Y-microspheres distribution in tumor and non-tumor based on ^99m^Tc-MAA SPECT/CT improves TARE efficacy [41, 42]. However, recent studies have challenged the reliability of the Y^90^ distribution in the liver [43–45]. This could be another reason for the lower mean tumor absorbed doses in the post-treatment dosimetry.

The results show that even 1-min scan might be sufficient for reliable post-treatment dosimetry evaluationfor the majority of cases with standard injected activities of over 1 GBq. However, this needs to be further investigated and variable uptake concentrations evaluated. In general, for dosimetry,a 5-min scan seems to be the shortest possible scan time for the general patient population with a large variety of injected activites. For image quality with 20-min equivalent SNR, a 5-min scan is recommended. This could facilitate the clinical planning without alternating the normal routine patient planning as well as the patient comfortability.

In comparison, another study [46] has shown that silicon photomultiplier (SiPM) PET/CT with AFOV of 20 cm produced 5- min images with sufficient image quality and enough count statistics for dosimetric post-treatment verification of the tumor absorbed dose.

The feasibility of lung dosimetry post-radioembolization within the same scan and scan time, simultaneously with the liver (which is not the case with standard AFOV scanners that may introduce motion and positioning mis-registration) with a whole-body scanner is clinically important. Our results show that the ^99m^Tc-MAA based predictive dosimetry calculates an extremely conservative mean lung absorbed dose, that is higher than the actual one. This may hinder unnecessarily the best treatment plan by a suboptimal Y90 injected activity, as two other studies using either SPECT/CT or PET/CT have already shown [47, 48].

## Conclusion

In this study we evaluated, in terms of image quality and dosimetry, whole-body PET clinical images of patients after having been treated with ^90^Y microspheres radioembolization for liver cancer. Compared to the 20-min standard scan, the simulated 5-min reconstructed images provided equal image peak SNR and noise bevahiour, while performing also similarly for post-treatment dosimetry of tumor, whole liver and lung absorbed doses.

### Electronic supplementary material

Below is the link to the electronic supplementary material.

Supplementary Material 1

Supplementary Material 2

Supplementary Material 3

Supplementary Material 4

Supplementary Material 5

Supplementary Material 6

Supplementary Material 7

Supplementary Material 8

## Declarations

*Research involving human participants and/or animals*.
