# Supplementary figures and images for: 90Y post-radioembolization clinical assessment with whole-body Biograph Vision Quadra PET/CT: image quality, tumor, liver and lung dosimetry
Source: Eur J Nucl Med Mol Imaging. 2024 Feb 13;51(7):2100–13. doi: 10.1007/s00259-024-06650-9 (PMC11139701; doi:10.1007/s00259-024-06650-9)

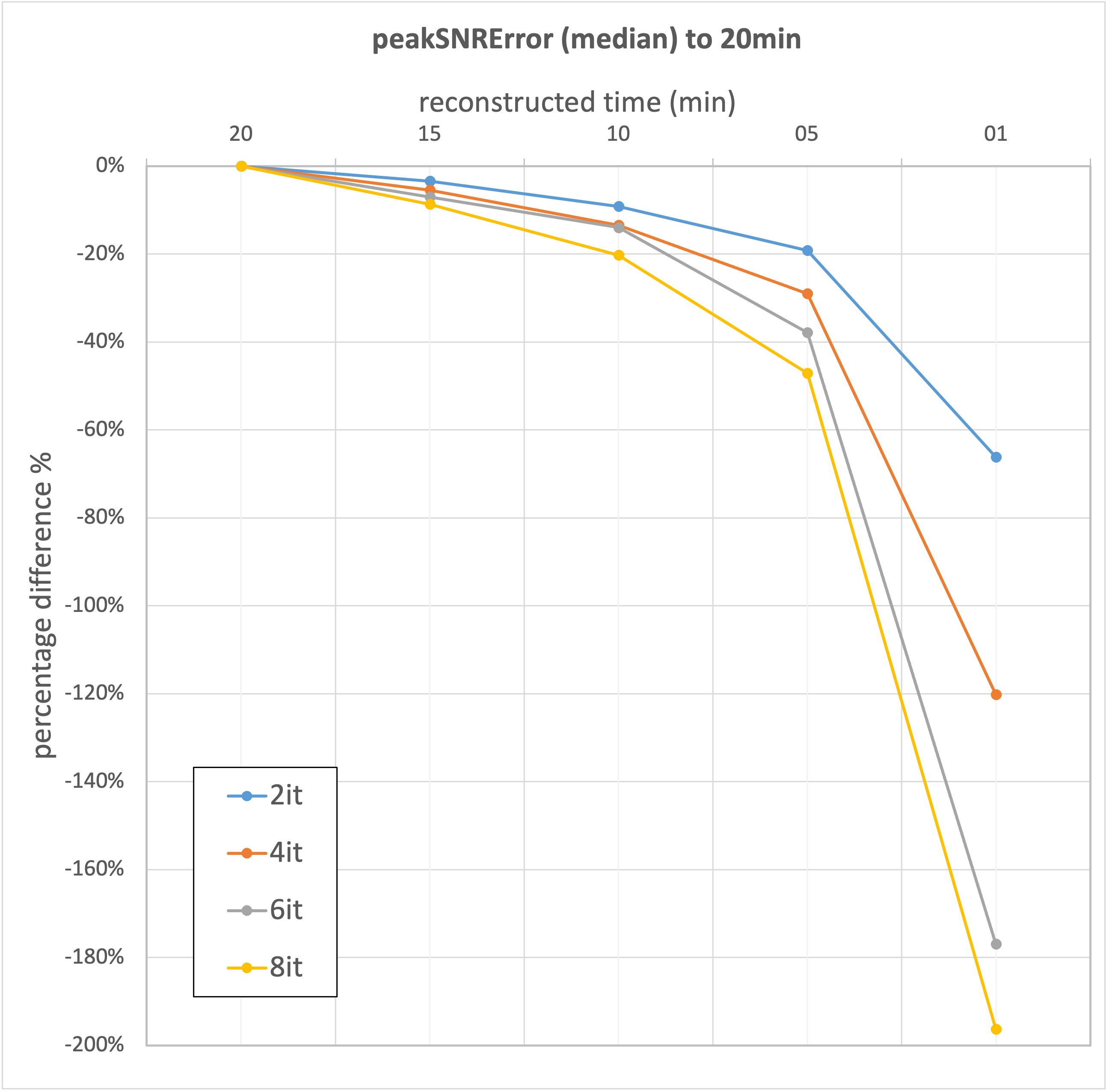

Supplement: Supplementary file 1 — Supplementary Material 1 [file 259_2024_6650_MOESM1_ESM.png]

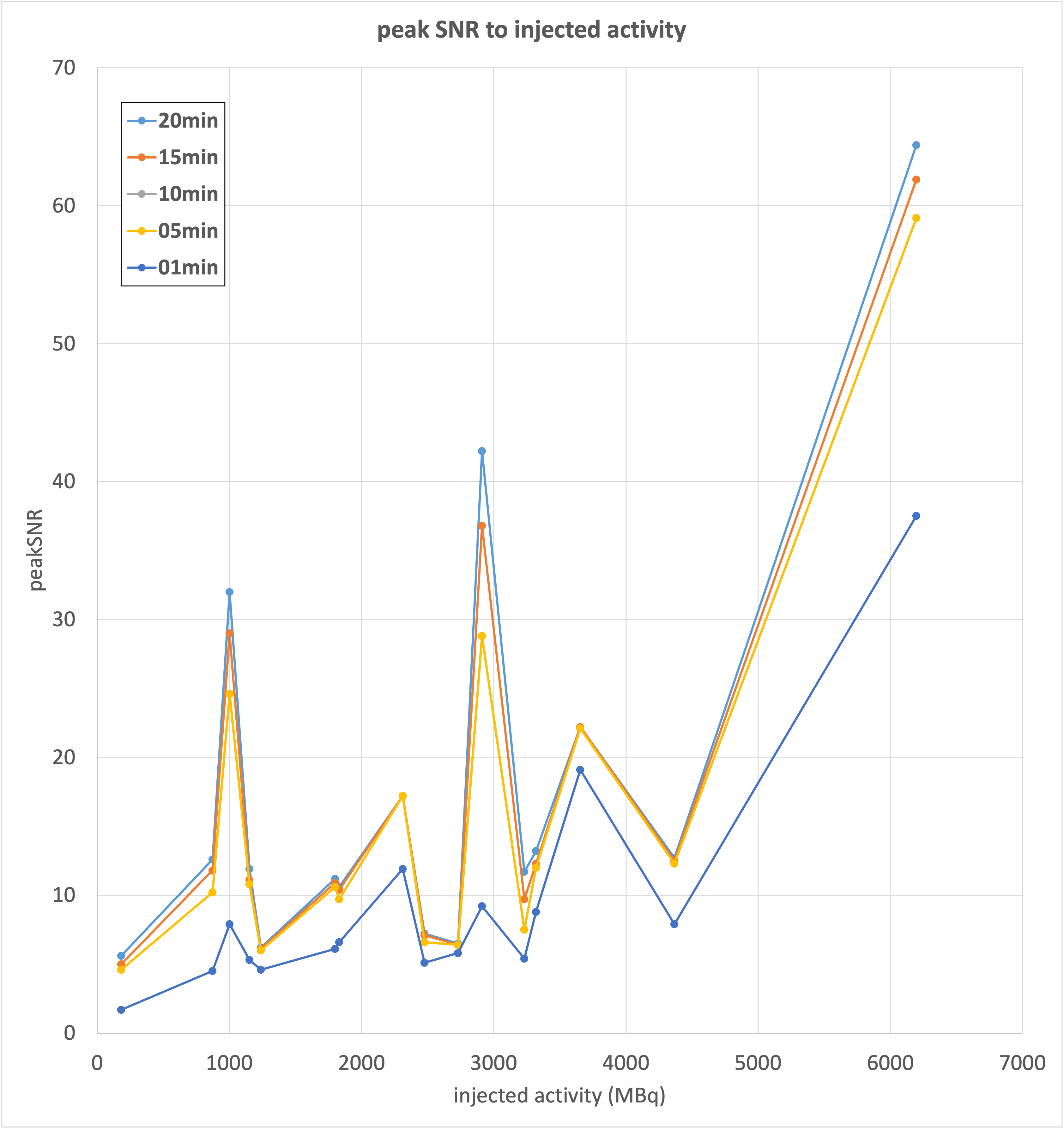

Supplement: Supplementary file 2 — Supplementary Material 2 [file 259_2024_6650_MOESM2_ESM.png]

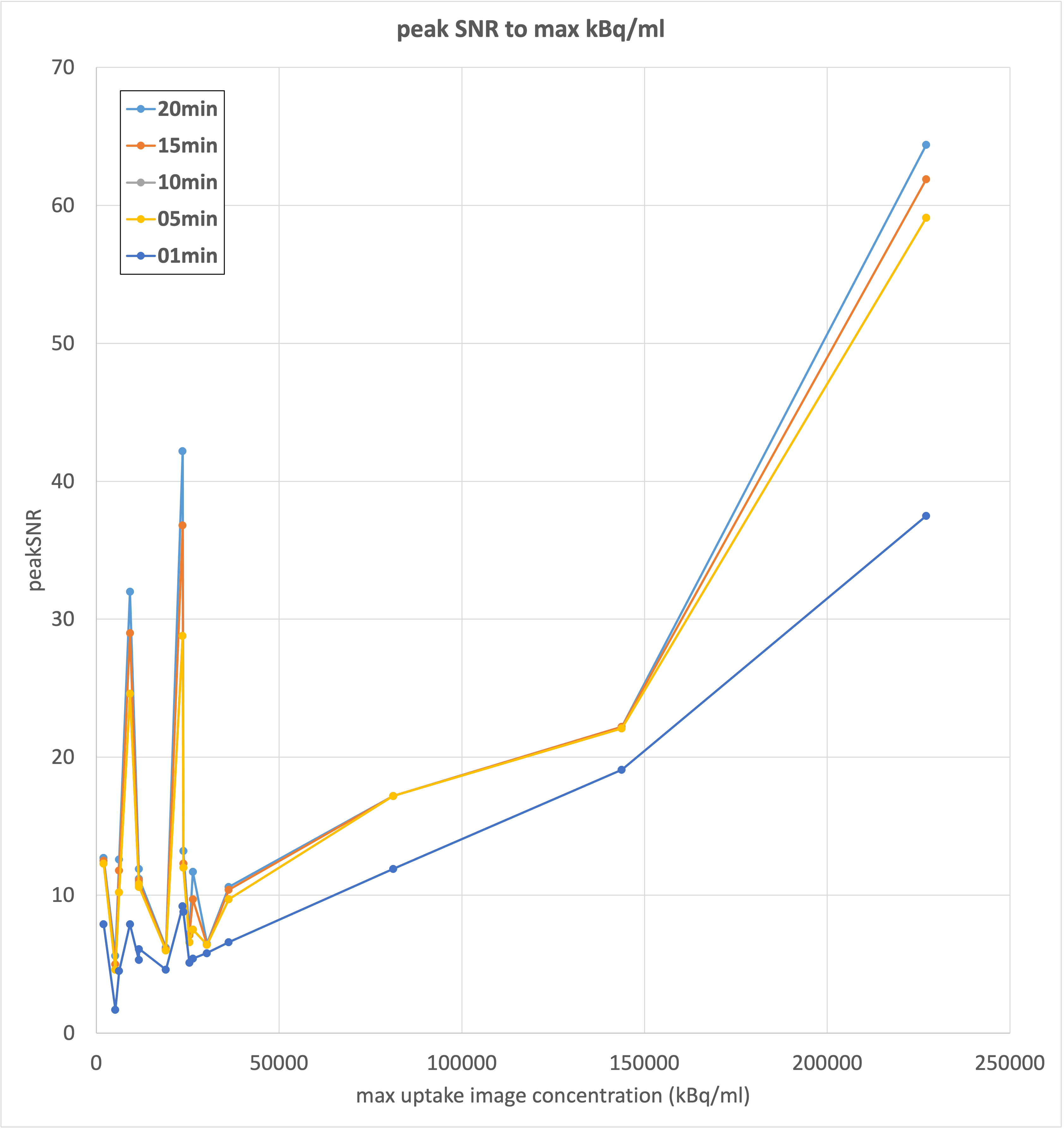

Supplement: Supplementary file 3 — Supplementary Material 3 [file 259_2024_6650_MOESM3_ESM.png]

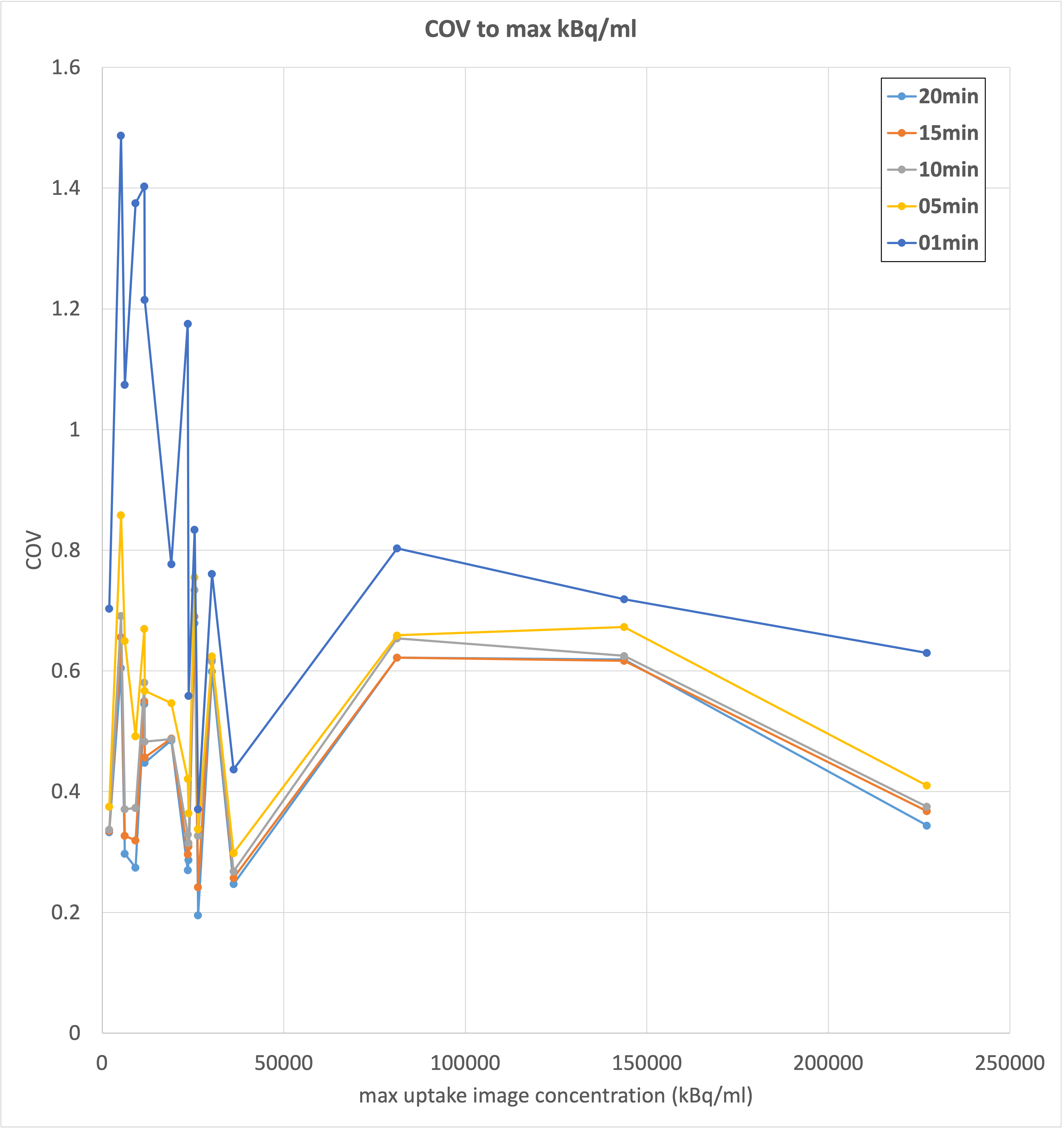

Supplement: Supplementary file 4 — Supplementary Material 4 [file 259_2024_6650_MOESM4_ESM.png]

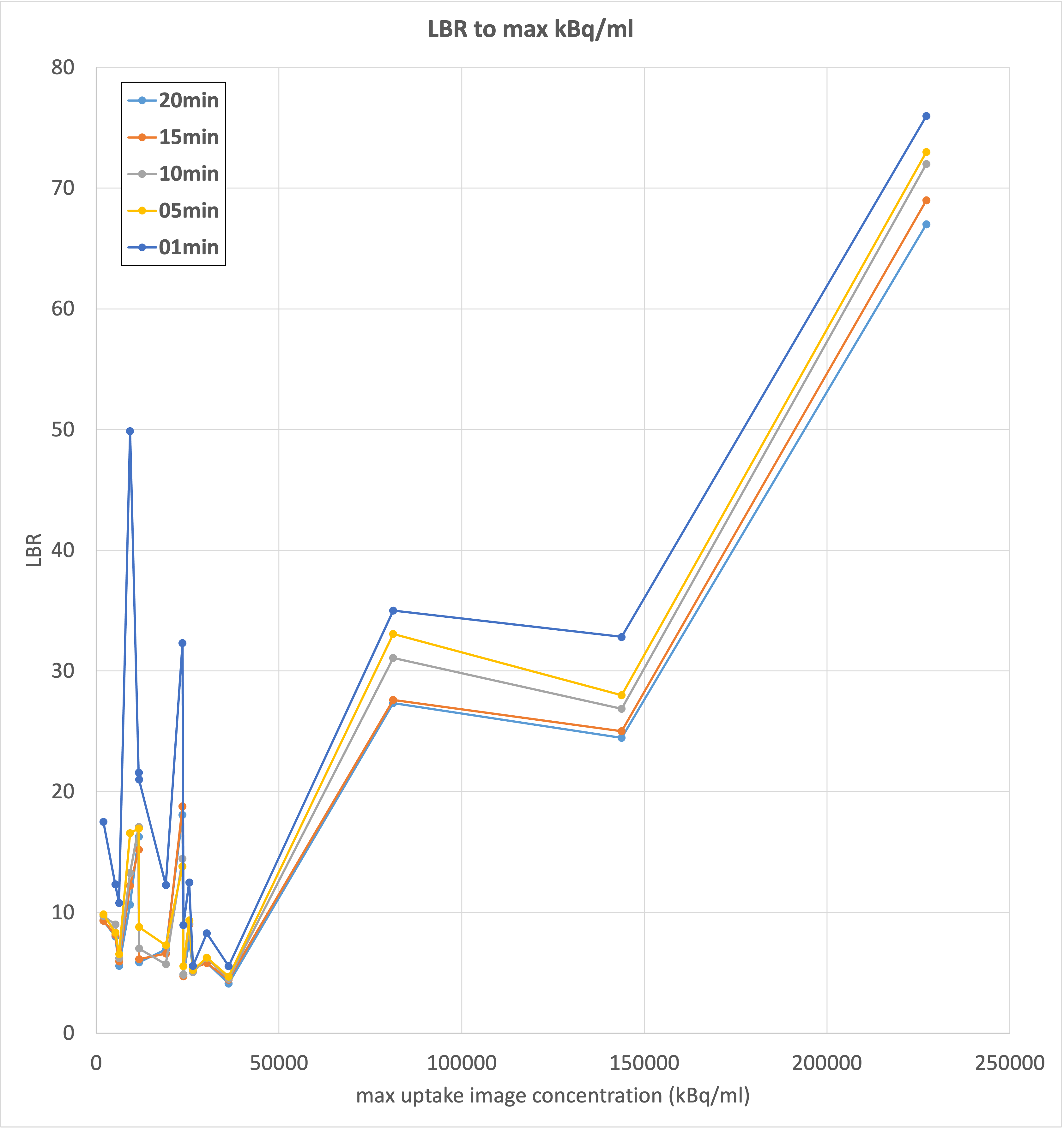

Supplement: Supplementary file 5 — Supplementary Material 5 [file 259_2024_6650_MOESM5_ESM.png]

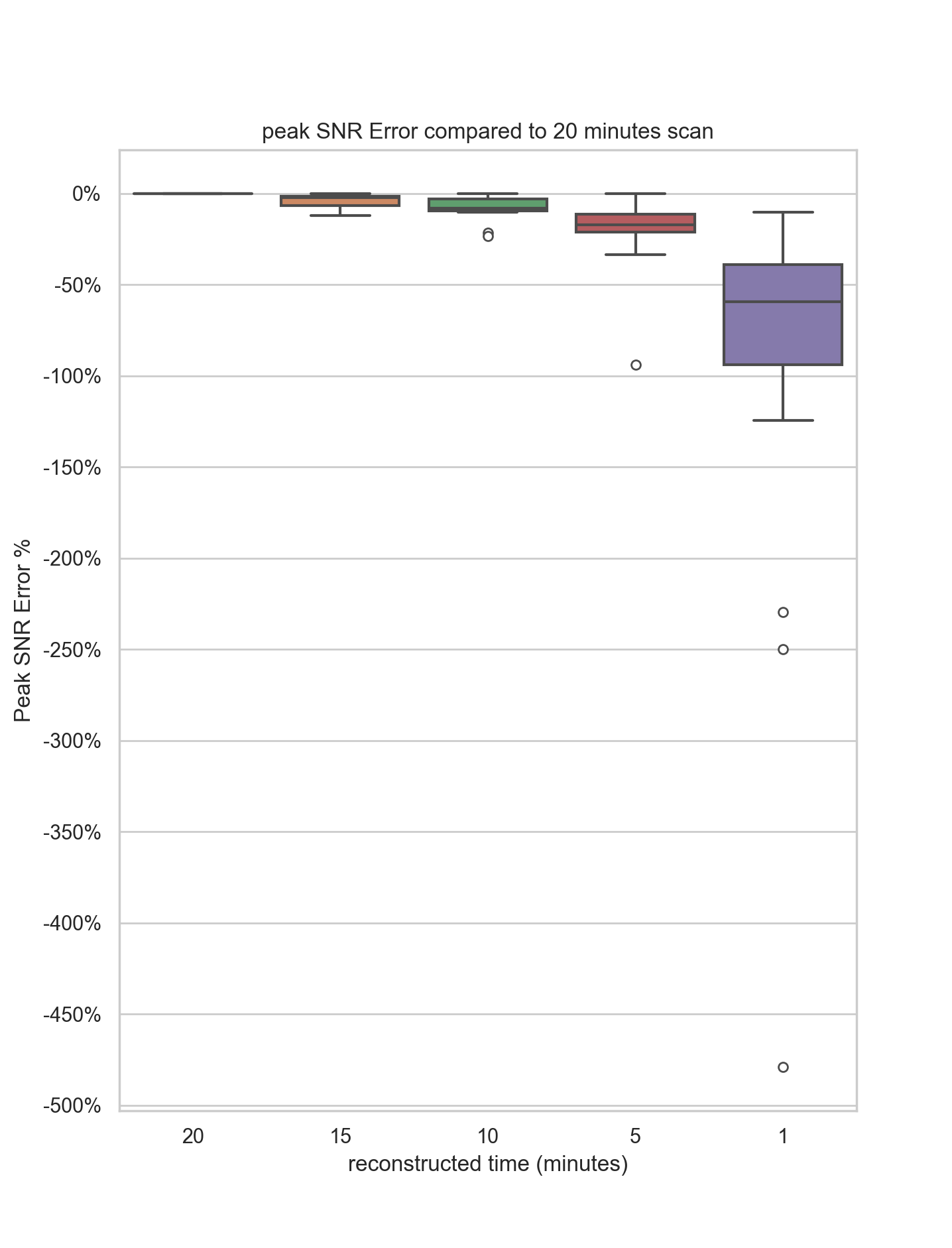

Supplement: Supplementary file 6 — Supplementary Material 6 [file 259_2024_6650_MOESM6_ESM.png]
