# Supplementary material for: 90Y post-radioembolization clinical assessment with whole-body Biograph Vision Quadra PET/CT: image quality, tumor, liver and lung dosimetry
Source: Eur J Nucl Med Mol Imaging. 2024 Feb 13;51(7):2100–13. doi: 10.1007/s00259-024-06650-9 (PMC11139701; doi:10.1007/s00259-024-06650-9)
Supplement: Supplementary file 7 — Supplementary Material 7 [file 259_2024_6650_MOESM7_ESM.docx]

| Injected activity (MBq) | Max. uptake concentration in image 20min2i (kBq/ml) |
| --- | --- |
| 181 | 5207 |
| 873 | 6240 |
| 1002 | 9205 |
| 1152 | 11623 |
| 1237 | 19050 |
| 1800 | 11695 |
| 1830 | 36198 |
| 2311 | 81226 |
| 2475 | 25453 |
| 2729 | 30226 |
| 2910 | 23580 |
| 3232 | 26407 |
| 3319 | 23819 |
| 3655 | 143753 |
| 4367 | 1944 |
| 6198 | 227117 |

**Table 1.** Injected activity to patients and corresponding maximum uptake concentration in the image reconstructed with 20 minutes and 2 iterations.
