# Supplementary material for: 90Y post-radioembolization clinical assessment with whole-body Biograph Vision Quadra PET/CT: image quality, tumor, liver and lung dosimetry
Source: Eur J Nucl Med Mol Imaging. 2024 Feb 13;51(7):2100–13. doi: 10.1007/s00259-024-06650-9 (PMC11139701; doi:10.1007/s00259-024-06650-9)
Supplement: Supplementary file 8 — Supplementary Material 8 [file 259_2024_6650_MOESM8_ESM.docx]

| **Reconstructed time (min)** | **01** | **05** | **10** | **15** | **20** |
| --- | --- | --- | --- | --- | --- |
|  | 473.3 | 450.3 | 459.5 | 466.6 | 441 |
|  | 118.9 | 130.9 | 140.4 | 145.9 | 144.1 |
|  | 256.1 | 272.3 | 268.5 | 271.1 | 253.5 |
|  | 269.9 | 265.6 | 267 | 266.6 | 265.5 |
|  | 87.8 | 93.3 | 97.1 | 99.7 | 100.7 |
|  | 67.7 | 86.2 | 90.2 | 89.2 | 90.1 |
|  | 106.5 | 96.2 | 89.7 | 89.2 | 87.3 |
|  | 334.4 | 296.2 | 292 | 291.7 | 292.5 |
|  | 236.4 | 227.2 | 221.8 | 220.6 | 218.6 |
|  | 345.1 | 322.3 | 316.7 | 314.1 | 312.3 |
|  | 307 | 302.2 | 306.9 | 310.6 | 311.6 |
|  | 402 | 403 | 399 | 401 | 400 |
|  | 394.3 | 378.1 | 357.6 | 348.7 | 353.3 |
|  | 666 | 511 | 511 | 513 | 511 |
|  | 109.2 | 112.3 | 112.4 | 110.9 | 111.4 |
|  | 666 | 511 | 511 | 513 | 511 |
|  | 199 | 217 | 226 | 230 | 232 |

**Table 2.** Abdorbed tumor doses for reconstructed times of 1 up to 20 minutes for all 17 patients.

| Percentage difference of absorbed tumor dose between 20minutes and 1minute reconstructions (cases with overestimation) | Percentage difference of absorbed tumor dose between 20minutes and 1minute reconstructions (cases with underestimation) | Mean, median, max, min values (cases with overestimation) | Mean, median, max, min values (cases with underestimation) |
| --- | --- | --- | --- |
| 7% | -21% | 10% | -14% |
| 1% | -15% | 10% | -15% |
| 2% | -33% | 23% | -1% |
| 18% | -1% | 0% | -33% |
| 13% | -2% |  |  |
| 8% |  |  |  |
| 10% |  |  |  |
| 0% |  |  |  |
| 10% |  |  |  |
| 23% |  |  |  |
| 23% |  |  |  |

**Table 3.** Percentage differences for the absorbed tumor dose between 20 minute and 1 minute reconstructions with their corresponding statistical metrics.


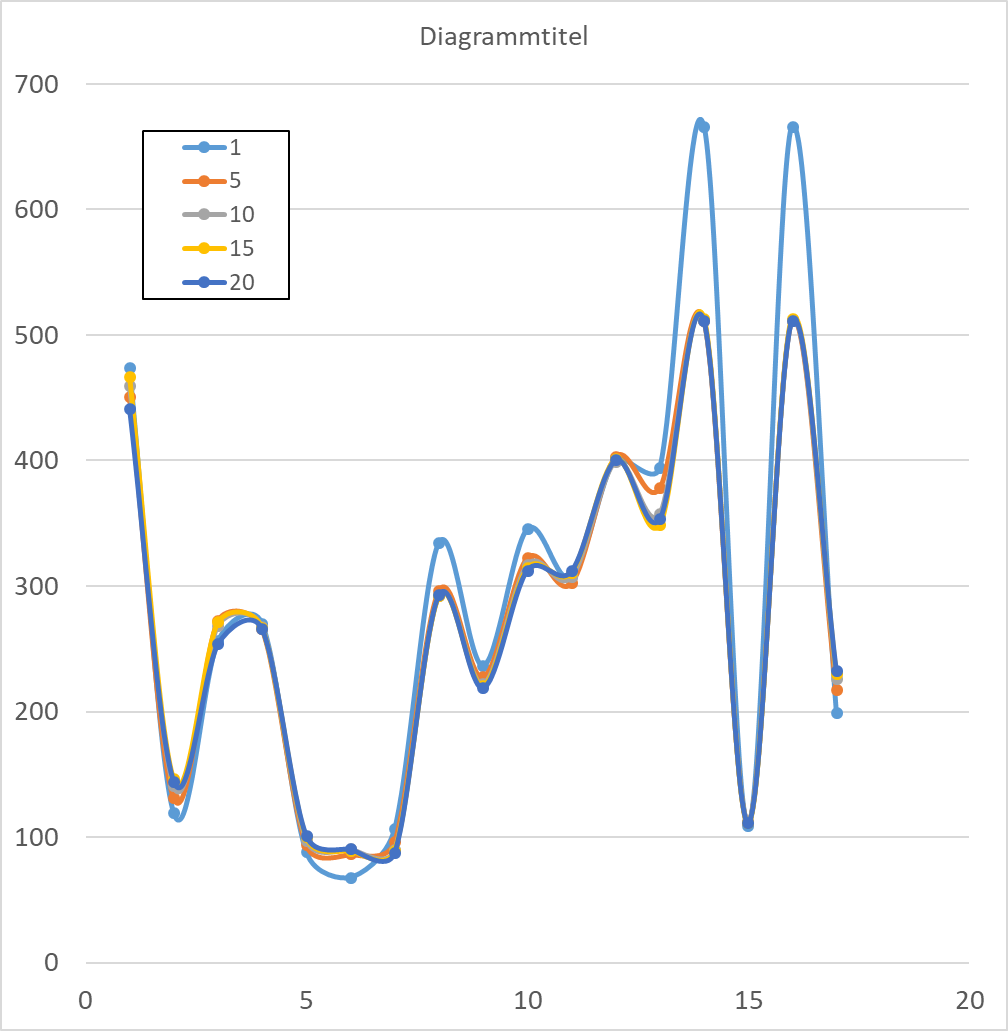


**Fig.1** Absorbed tumor doses for all patients (x-axis) for all reconstructions.
